# Efficient Removal of Ciprofloxacin from Water Using High-Surface-Area Activated Carbon Derived from Rice Husks: Adsorption Isotherms, Kinetics, and Thermodynamic Evaluation

**DOI:** 10.3390/molecules30122501

**Published:** 2025-06-07

**Authors:** Esra Demirdağ, Mehmet Ferit Demirel, Veysel Benek, Elif Doğru, Yunus Önal, Mehmet Hüseyin Alkan, Kadir Erol, İhsan Alacabey

**Affiliations:** 1 The Institute of Science, Department of Chemistry, Dicle University, 21280 Diyarbakır, Turkey; esrademirdag2@gmail.com; 2 Department of Medical Services and Techniques, Vocational School of Health Services, Artuklu University, 47100 Mardin, Turkey; mehmetferit@artuklu.edu.tr (M.F.D.); ihsanalacabey@hotmail.com (İ.A.); 3 Vakıfbank Secondary School, Ministry of National Education, 65300 Van, Turkey; vbenek@yahoo.com; 4 Department of Chemistry, The Institute for Graduate Educational Studies, Artuklu University, 47100 Mardin, Turkey; elifcann10@gmail.com; 5 Department of Chemical Engineering, Faculty of Engineering, Inonu University, 44280 Malatya, Turkey; yunus.onal@inonu.edu.tr; 6 Department of Basic Pharmaceutical Sciences, Faculty of Pharmacy, Dicle University, 21280 Diyarbakır, Turkey; 7 Department of Medical Services and Techniques, Vocational School of Health Services, Hitit University, 19030 Corum, Turkey

**Keywords:** activated carbon, adsorption, antibiotic pollution, ciprofloxacin, rice husk

## Abstract

Activated carbon is widely recognized as an effective material for removing pollutants, especially pharmaceutical residues, from water. In this study, high-surface-area activated carbon derived from rice husks (RHAC) was synthesized via KOH activation and used for the adsorption of ciprofloxacin, a widely used fluoroquinolone antibiotic. Its adsorption behavior was systematically investigated through batch experiments varying the pH, adsorbent dosage, contact time, initial concentration, and temperature. The RHAC exhibited a high surface area of 1539.7 m^2^/g and achieved a maximum adsorption capacity of 398.4 mg·g^−1^. The Freundlich isotherm best describes its adsorption equilibrium, suggesting multilayer adsorption on a heterogeneous surface. Kinetic modeling revealed that the adsorption process followed a pseudo second-order model (R^2^ = 0.9981), indicating chemisorption as the rate-limiting mechanism. Thermodynamic parameters (ΔH° = 6.61 kJ/mol, ΔG° < 0) confirmed that the process was endothermic and spontaneous. These findings demonstrate that RHAC is a highly efficient, low-cost, and sustainable adsorbent for removing ciprofloxacin from aqueous environments.

## 1. Introduction

With the development of technology and the increase in population, the use of chemicals has increased, and the pollution of surface and groundwater is becoming an increasingly severe problem worldwide [1,2]. Chemicals that come from sources such as industrial waste, agricultural activities, domestic waste, petroleum products, and pharmaceutical waste cause water pollution [3]. Water resources have decreased worldwide and in our country due to global warming over the last century. Given the scarcity of clean water, numerous studies have focused on developing effective wastewater treatment methods [4,5,6].

Antibiotics, while essential in fighting infections, pose significant environmental risks due to their persistence in aquatic environments [7]. The problem lies in their incomplete metabolism in our bodies, leading to their excretion and eventual entry into water bodies [8]. The presence of these drug residues in the aquatic environment is a major contributor to the development of antibiotic resistance and may induce acute toxicological effects on the surrounding organisms [9,10].

Ciprofloxacin (Cipro) is a widely consumed broad-spectrum antimicrobial from the fluoroquinolone group, extensively used in both human and veterinary medicine due to its potent antibacterial activity [11,12]. According to pharmacokinetic data, 55% of oral Cipro is excreted in urine after 4 h. Due to its frequent use, persistent high concentrations of Cipro and other antibiotics are found in wastewater. If these antibiotics are not adequately treated, they reach our drinking water, which causes various adverse effects on human health [13].

Various methods, such as biological treatment, photocatalytic degradation, ozonation, and membrane filtration, remove pharmaceutical substances from wastewater [14,15]. The adsorption technique, which is easy to apply and more economical, is mainly preferred [16,17]. Activated carbon, with its large surface area and porous structure, is an adsorbent used to remove various water pollutants, including different pharmaceuticals [18,19]. However, the adsorption of antibiotics by activated carbon varies depending on the activated carbon source used, production process, and adsorbent structure [20,21].

In our study, activated carbon was obtained using rice husks, and then the removal of Cipro from aqueous solutions with the activated carbon was assessed. This study presents a novel and sustainable approach to removing Cipro, a persistent pharmaceutical contaminant, from aqueous solutions using activated carbon derived from rice husks. The innovation of this study lies in the environmentally friendly and cost-effective transformation of rice husks, an abundantly available agricultural waste product, into high-surface-area activated carbon through a scalable potassium hydroxide (KOH) activation process. This sustainable method valorizes a low-value byproduct and eliminates the need for harsh chemical treatments typically used in conventional activation techniques. By aligning with the principles of green chemistry and the circular economy, this approach reduces waste generation, enhances resource efficiency, and contributes to the development of affordable and effective adsorbents for water purification applications. pH, adsorbent amount, initial Cipro concentration, and temperature parameters were investigated for optimum adsorption conditions. Calculations were made by applying Langmuir, Dubinin–Radushkevich (D–R), Freundlich, and Temkin adsorption isotherm equations.

## 2. Results and Discussion

### 2.1. Characterization

**Figure 1:**
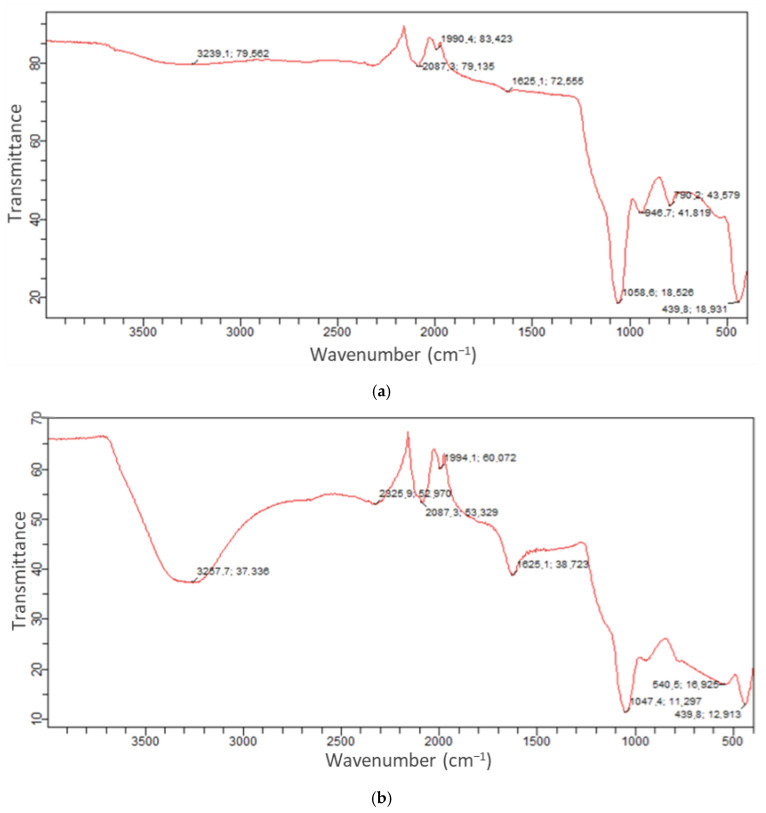
FTIR spectrum of (**a**) RHAC, (**b**) Cipro-loaded RHAC.

The FT-IR spectra of KOH-activated rice husk-derived activated carbon (Figure 1a) and Cipro-loaded activated carbon (Figure 1b) reveal significant changes that confirm the successful adsorption of Cipro onto the activated carbon surface.

In the bare activated carbon spectrum, a broad peak observed around 3239 cm^−1^ corresponds to O–H stretching vibrations, indicating the presence of surface hydroxyl groups or adsorbed water (Figure 1a). Peaks at 2087 cm^−1^ and 1990 cm^−1^ may be attributed to C≡C or C≡N stretching vibrations, typical of activated carbon structures with residual carbon species. The peak at 1625 cm^−1^ is associated with the C=C stretching of aromatic rings, reflecting the carbonaceous structure [22,23]. Peaks observed in the fingerprint region (around 1058 cm^−1^, 947 cm^−1^, and 439 cm^−1^) can be attributed to C–O stretching, deformation vibrations, and Si–O–Si bonds, respectively, which are commonly found in rice husk-based materials due to their natural silica content [24].

Several essential spectral changes are evident in the Cipro-loaded activated carbon spectrum (Figure 1b). Firstly, the O–H stretching band shifts and reduces in intensity (from 3239 cm^−1^ to 3267 cm^−1^), suggesting hydrogen bonding interactions between the hydroxyl groups of the activated carbon and Cipro. Additionally, new or enhanced bands appear around 2087 cm^−1^, 1994 cm^−1^, and 1625 cm^−1^, which may correspond to the characteristic functional groups of Cipro, such as carboxylic C=O, aromatic C=C, and amide C=O stretching. Moreover, the increased peak intensities at 947 cm^−1^ and 540 cm^−1^ suggest additional interactions or complexation between the drug molecules and the surface functionalities of activated carbon [25].

Additionally, FTIR spectral shifts (especially in the O–H stretching region and the appearance or intensification of bands associated with Cipro’s functional groups) support the presence of hydrogen bonding and electrostatic attractions. These spectral changes confirm that electrostatic forces play a crucial role in the adsorption mechanism under acidic conditions [26].

**Figure 2:**
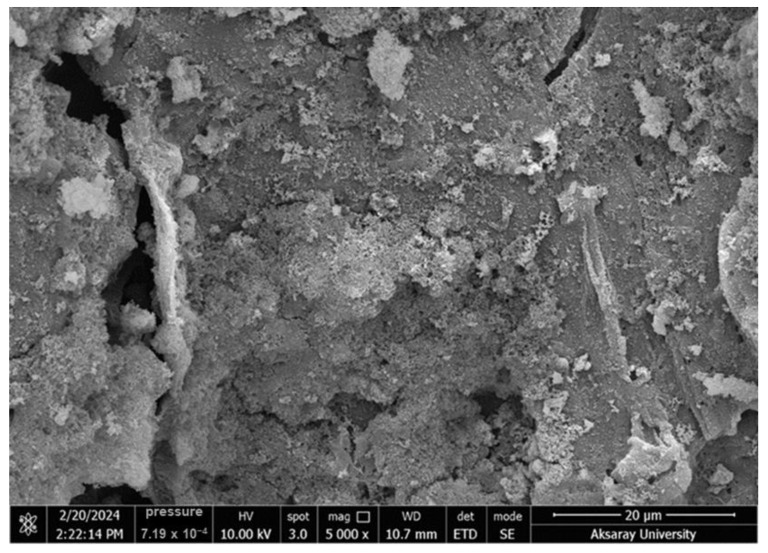

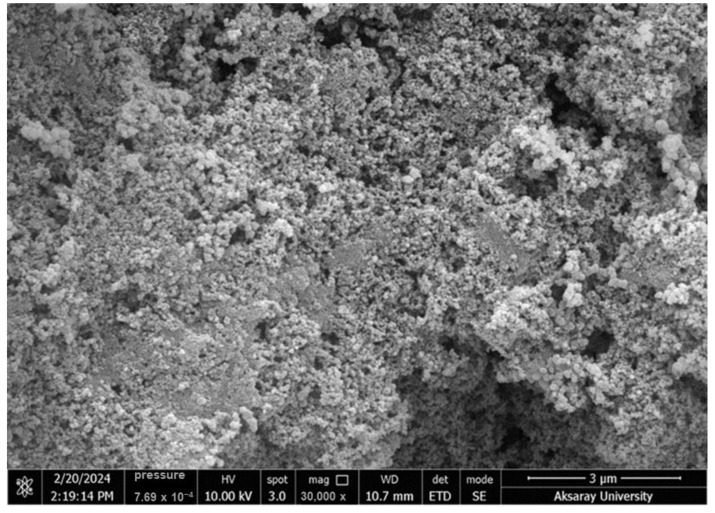
SEM images of RHAC.

Figure 2 displays SEM micrographs illustrating the surface morphology and pore structure of RHAC at two magnifications. The SEM images of rice husk-derived activated carbon at magnifications of 5000× and 30,000× reveal valuable insights into the surface morphology and porosity of the material. At 5000× magnification, the surface shows irregularities with visible cracks, cavities, and porous structures, indicating that the chemical activation process successfully developed a well-defined porous network. These features enhance the surface area and provide favorable sites for adsorbing pollutants. At a higher magnification of 30,000×, the surface is observed to be granular and heterogeneous, composed of fine, clustered particles and micro- to nano-scale pores. Such a morphology is attributed to thermal decomposition and the removal of intrinsic silica content. The interconnected pores and rough texture contribute significantly to the high specific surface area of the activated carbon, making it a highly effective adsorbent for environmental applications such as removing antibiotics from aqueous solutions.

The specific surface area of the rice husk-derived activated carbon was found to be 1539.7 m^2^/g, indicating the successful development of a highly porous structure through the activation process. Such a high surface area is a critical parameter for adsorption applications, as it directly correlates with the number of available active sites on the adsorbent surface. This value suggests that the chemical activation effectively created a network of micro- and mesopores, enhancing the material’s ability to interact with and capture pollutant molecules like Cipro. Activated carbon materials with surface areas exceeding 1000 m^2^/g are generally considered highly efficient in environmental remediation, particularly in removing pharmaceuticals and other organic contaminants from aqueous solutions [27,28]. Therefore, the measured surface area strongly supports the potential of this material as an excellent adsorbent in wastewater treatment applications [29].

**Figure 3:**
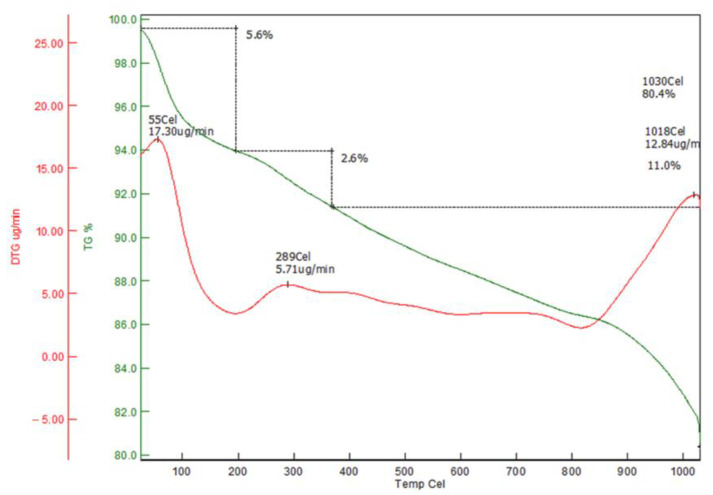
DTA-TGA curve of RHAC.

Figure 3 presents the DTA-TGA thermal profile of RHAC, highlighting its decomposition stages and thermal stability. The TGA-DTG analysis reveals the sample’s thermal behavior and decomposition pattern across a temperature range of up to 1030 °C. The initial weight loss of approximately 5.6% observed around 55 °C is attributed to the evaporation of surface and physically adsorbed moisture, as indicated by a sharp DTG peak at this temperature (17.30 µg/min). A second, more minor weight loss of 2.4% occurs between 100 °C and 400 °C, corresponding to the degradation of low-molecular-weight organic compounds, with a DTG peak at 289 °C (5.71 µg/min). From 400 °C to 800 °C, the TGA curve remains relatively stable, suggesting high thermal stability of the material in this range. A further significant weight loss of about 11% occurs between 800 °C and 1030 °C, with marked decomposition peaks at 1018 °C (12.84 µg/min) and 1030 °C, indicating the breakdown of carbon-rich structures. Overall, the material possesses a total weight loss of approximately 19%, demonstrating a multi-step decomposition process involving moisture loss, organic degradation, and carbon matrix decomposition at elevated temperatures.

### 2.2. Adsorption Experiments

**Figure 4:**
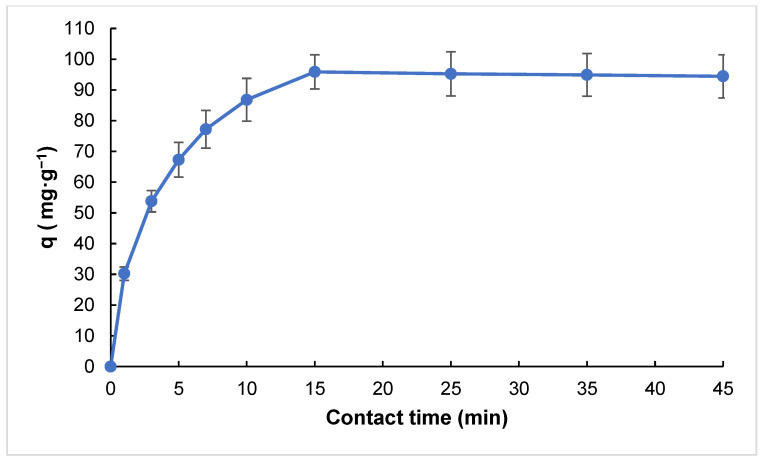
Effect of contact time on adsorption capacity (pH: 7.0, amount of adsorbent: 50 mg, adsorbate concentration: 50 mg·L^−1^, V: 0.10 L, T: 298 K).

The optimum contact time was determined by plotting the adsorption capacity as a function of time during the experimental studies. As shown in Figure 4, the adsorption process occurs rapidly on the RHAC surface during the initial stages. However, this rate gradually decreases over time and levels off after approximately 15 min. This trend suggests rapid saturation of the available active sites on the RHAC surface within the first 15 min [30]. Therefore, this duration was chosen as the optimum contact time, and all subsequent adsorption experiments were carried out accordingly.

**Figure 5:**
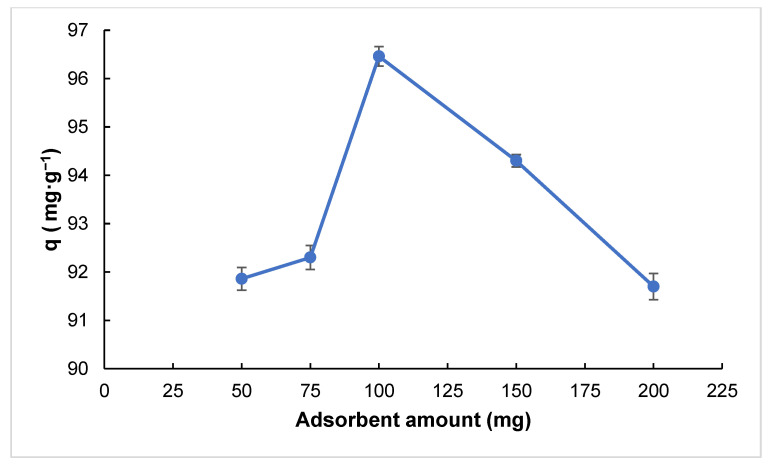
Effect of adsorbent amount on adsorption capacity (pH: 7.0, contact time: 15 min, adsorbate concentration: 50 mg·L^−1^, V: 0.1 L, T: 298 K).

The graph shows that the adsorption capacity per gram of activated carbon (*q*) increases with increasing adsorbent amount, reaching a maximum of around 100 mg (Figure 5). At lower adsorbent doses, the number of available active sites is insufficient to adsorb all Cipro molecules in the solution, resulting in lower *q* values. As the adsorbent amount increases to an optimal level, more active sites become available, leading to higher adsorption efficiency. However, beyond 100 mg, the adsorption capacity per gram decreases. This phenomenon can be attributed to the distribution of a fixed amount of Cipro molecules over a larger adsorbent surface, leading to underutilized active sites [31]. Excessive adsorbent may lead to particle agglomeration, which can block pores and reduce the effective surface area [32]. Accordingly, 100 mg was identified as the optimum adsorbent dosage under the given experimental conditions.

**Figure 6:**
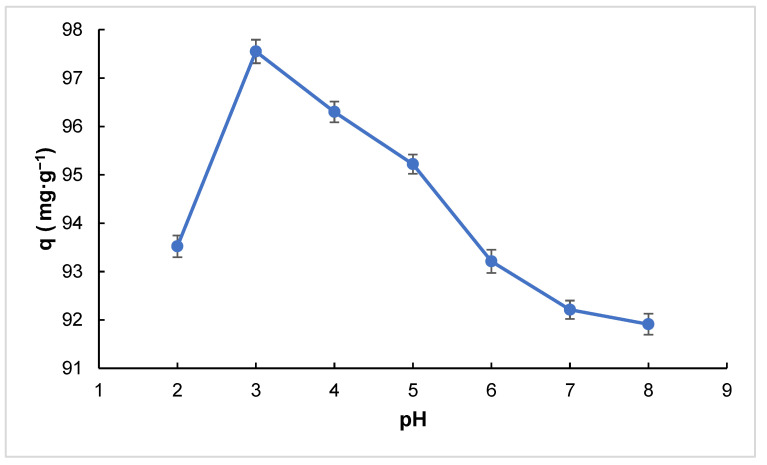
Effect of pH on adsorption capacity (amount of adsorbent: 100 mg, contact time: 15 min, adsorbate concentration: 50 mg·L^−1^, V: 0.1 L, T: 298 K).

The adsorption capacity of Cipro onto RHAC was found to be highest at pH 3.0, as shown in the graph (Figure 6). Considering that the isoelectric point (pI) of RHAC lies between pH 7.0 and 8.0 [22,33], this result can be attributed to the electrostatic interactions between the adsorbent and the adsorbate. At low pH values, particularly around pH 3.0, the RHAC surface is positively charged. At the same time, Cipro exists primarily in a zwitterionic or partially cationic form due to the protonation of its functional groups. This form leads to a strong electrostatic attraction between the negatively charged carboxylate groups on the positively charged RHAC surface and Cipro’s positively charged amino groups, enhancing adsorption.

Additionally, low pH conditions reduce competition from other ions in the solution, promoting more efficient interactions between the adsorbent and the drug molecules. In contrast, at higher pH levels, the RHAC surface becomes negatively charged, and Cipro also tends to adopt a more anionic form. These conditions result in electrostatic repulsion, reducing the overall adsorption capacity. Moreover, the presence of excess OH^−^ ions at higher pH values can compete with Cipro molecules for active sites, further decreasing adsorption. Hence, the optimal adsorption observed at pH 3.0 highlights the significance of electrostatic attraction in the adsorption mechanism under acidic conditions.

**Figure 7:**
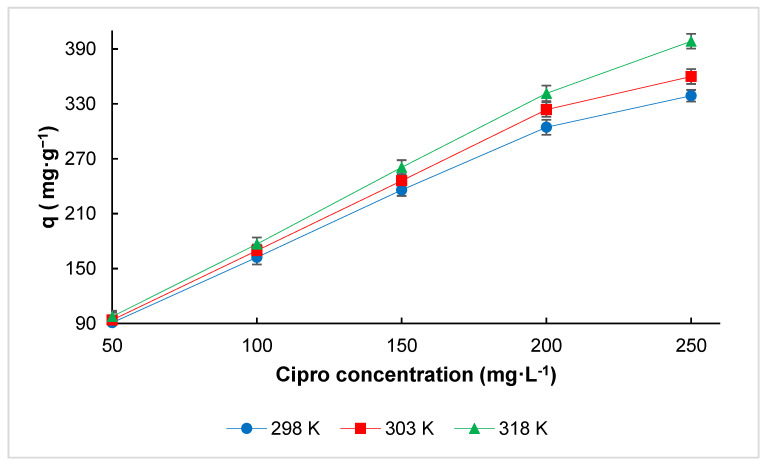
Effect of temperature on the adsorption capacity of RHAC for Cipro at 298 K, 303 K, and 318 K. Each data point represents the mean of three independent parallel experiments, and the error bars indicate the standard deviation (SD) (pH: 3.0, amount of adsorbent: 100 mg, contact time: 15 min, V: 0.1 L).

The graph illustrates the adsorption capacity of RHAC for Cipro at different concentrations and temperatures (298 K, 303 K, and 318 K). As the concentration of Cipro increases from 50 to 250 mg·L^−1^, the adsorption capacity also increases consistently across all temperatures (Figure 7). Notably, higher temperatures result in greater adsorption capacities, indicating that the adsorption process is endothermic [34]. The highest adsorption is observed at 318 K, followed by 303 K and 298 K. In addition, the interaction forces between the solvent and the solute increased further due to the observed increase in the solubility of Cipro, thus making it more challenging to adsorb the solute [35].

**Figure 8:**
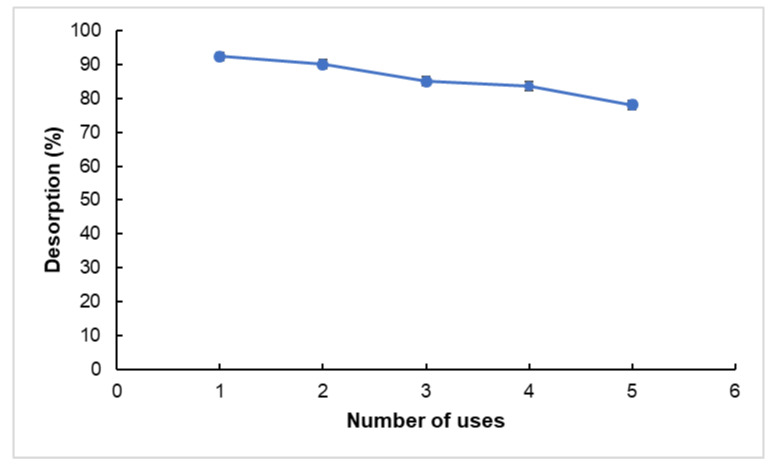
Desorption efficiency and reusability performance of the adsorbent over multiple cycles (amount of adsorbent: 100 mg, contact time: 120 min, adsorbate concentration: 50 mg·L^−1^, V: 0.1 L, T: 298 K).

The reusability performance of RHAC was evaluated over five consecutive adsorption/desorption cycles using Cipro (Figure 8). Desorption was carried out by treating the exhausted adsorbent with a 0.1 M NaOH solution under gentle agitation for 2 h. After each cycle, the regenerated RHAC was washed thoroughly, dried, and reused for subsequent Cipro adsorption.

The adsorption efficiencies for cycles 1 through 5 were determined to be 92.45%, 90.14%, 85.12%, 83.62%, and 78.12%, respectively. These results indicate a gradual decline in adsorption capacity, with a total efficiency loss of approximately 15.4% after five cycles. Despite this reduction, the RHAC retained over 78% of its initial performance, suggesting satisfactory regeneration and structural stability of the adsorbent under alkaline desorption conditions.

The observed decrease in adsorption capacity over repeated use can be attributed to the incomplete removal of Cipro molecules during desorption, partial pore blockage, or potential alteration of surface functional groups. However, the relatively high efficiency retention is consistent with findings reported in similar studies, where over 75–85% of adsorption capacity was maintained after multiple regeneration cycles [36,37].

The adsorption performance (pH 3.0, 100 mg adsorbent dosage, 15 min contact time, and room temperature conditions) of RHAC in simulated hospital wastewater was notably lower than that observed in distilled water media. Specifically, its adsorption capacity decreased from 90.81 mg·g^−1^ to 51.38 mg·g^−1^ at 50 mg·L^−1^, and from 338.90 mg·g^−1^ to 213.24 mg·g^−1^ at 250 mg·L^−1^. This reduction is likely attributed to the complex matrix of real wastewater, which includes competing organic and inorganic constituents, such as other pharmaceutical residues, ions, and natural organic matter, that may interfere with the active binding sites of the RHAC [38]. Moreover, the presence of electrolytes and particulate matter in hospital effluents may contribute to partial pore blockage or surface fouling, further reducing the available adsorption capacity [39]. Nevertheless, the RHAC still demonstrated substantial Cipro uptake, confirming its potential applicability for real-world water treatment systems. These findings emphasize the necessity of conducting adsorption studies in environmentally relevant matrices to predict field-scale performance better and optimize operational parameters accordingly.

### 2.3. Adsorption Isotherms

Adsorption isotherms are mathematical models that describe the distribution of adsorbate species between liquid and solid phases. The Freundlich, Langmuir, Temkin, and Dubinin–Radushkevich (D-R) isotherm equations were used to determine Cipro’s adsorption mechanisms on activated carbon.

Substances exhibit a specific distribution in the solid and liquid phases at equilibrium. The ratio of species in the liquid and solid phases measures the equilibrium phenomenon during adsorption. The amount adsorbed per unit of adsorbent is plotted against the equilibrium adsorption concentration remaining in the solution at a constant temperature to express the adsorption equilibrium. These curves, which are generally nonlinear, are called adsorption isotherms.

Many researchers have used Langmuir and Freundlich isotherms to explain the adsorption mechanism [40,41]. In addition, the D-R adsorption isotherm model, which is a more general model than the Freundlich and Langmuir models, is also applied. Since these models do not account for temperature effects, the Temkin model was applied to evaluate the influence of thermal conditions. This study was repeated separately at 298 K, 303 K, and 318 K. The isotherm constants calculated from the linear graphs drawn using the Langmuir, Freundlich, Dubinin–Radushkevich, and Temkin isotherm models are given in Table 1. When the isotherm parameters calculated in Table 1 are examined, it is seen that the best correlation for Cipro adsorption on RHAC is better suited to the Freundlich isotherm. Equations for calculating the parameters and detailed comments are presented in the Supplemental Material.

### 2.4. Kinetic Modeling

Kinetic models are essential for understanding the adsorption process’s mechanisms, especially in identifying whether physical or chemical interactions predominate. The pseudo first-order and pseudo second-order models are the most widely used to describe adsorption kinetics. The pseudo first-order model, proposed by Lagergren, assumes that the occupation rate of adsorption sites is directly proportional to the number of unoccupied sites. This model typically applies to systems where physical adsorption (such as van der Waals forces) is the dominant mechanism.

The linear form of the model is given by:

(1) $$\log\left(q_{e}-q_{t}\right)=\log q_{e}-\left(\frac{k_{1}}{2.303}\right)t$$

where *q_t_* is the amount of adsorbate adsorbed at time *t* (mg·g^−1^), *q_e_* is the amount adsorbed at equilibrium (mg·g^−1^), and *k*_1_ is the pseudo first-order rate constant (1/min). A linear plot of *log*(*q_e_* − *q_t_*) versus *t* would indicate conformity to the pseudo first-order kinetic model. From the slope and intercept of the linear plot, the rate constant *k*_1_ and the theoretical equilibrium capacity *q_e_* can be determined.

The pseudo second-order model, proposed by Ho and McKay, assumes that the adsorption follows a second-order reaction mechanism, where the rate-limiting step involves chemical adsorption (chemisorption) through the sharing or exchange of electrons between adsorbent and adsorbate.

The linear form of the model is:

(2) $$\frac{t}{q_{t}}=\frac{1}{k_{2}{q_{e}}^{2}}+\frac{1}{q_{e}}t$$

where *k*_2_ is the pseudo second-order rate constant (g/mg·min). A plot of *t*/*q_t_* against *t* yields a straight line if the system follows this model. The slope and intercept can be used to calculate the equilibrium adsorption capacity *q_e_* and the rate constant *k*_2_.

Since the pseudo first-order model often fails to predict the experimental *q_e_* accurately and results in lower correlation coefficients (R^2^), the pseudo second-order model generally provides a better fit, particularly in systems with rapid adsorption and higher capacities. A strong agreement with the pseudo second-order model suggests that chemisorption is the dominant mechanism [42].

The adsorption kinetics of Cipro onto RHAC were analyzed using both pseudo first-order and pseudo second-order kinetic models (Table 2). The experimental data revealed a significantly better fit with the pseudo second-order model, which yielded a high correlation coefficient (R^2^ = 0.9981) compared to the pseudo first-order model (R^2^ = 0.8862). For the pseudo first-order model, the theoretical equilibrium adsorption capacity (*q_e_*) was calculated as 45.56 mg·g^−1^, which deviated considerably from the experimentally observed value (~95.1 mg·g^−1^). This mismatch indicates that the pseudo first-order model does not adequately describe the adsorption behavior in this system. In contrast, the pseudo second-order model estimated an equilibrium capacity of 100.15 mg·g^−1^, closely matching the experimental value. Furthermore, the model’s high correlation coefficient and consistent rate constant (k_2_ = 0.0051 g/mg·min) suggest that the adsorption process is likely governed by chemisorption mechanisms involving valence forces through the sharing or exchange of electrons between adsorbent and adsorbate.

These findings collectively support a pseudo second-order kinetic model, implicating chemisorption as the dominant adsorption mechanism.

### 2.5. Adsorption Thermodynamic Results

Adsorption thermodynamics is essential to determine how an adsorption process occurs and the mass transfer mechanism of the adsorbate to the adsorbent under equilibrium conditions. Adsorption thermodynamics also investigates whether the adsorption process is positive or negative at changing temperatures [43]. The thermodynamic behavior of the adsorption process is evaluated using parameters such as Gibbs free energy (ΔG°), enthalpy (ΔH°), and entropy (ΔS°). ΔG° indicates whether the process is spontaneous, while ΔH° reveals whether the adsorption is exothermic or endothermic. Additionally, ΔH° helps to distinguish whether the mechanism is physical (physisorption) or chemical (chemisorption). On the other hand, ΔS° provides insight into the degree of structural disorder at the solid–liquid interface and reflects whether the process is reversible, particularly when negative values are obtained [44]. The thermodynamic parameters calculated for the adsorption of Cipro on RHAC are given in Table 3. The parameters were determined using the Van’t Hoff equation based on dimensionless equilibrium constants derived from the experimental data. Detailed derivation steps, equilibrium constant normalization, and supporting thermodynamic plots are provided in the Supplementary Material to ensure transparency and reproducibility.

### 2.6. Literature Review

Various biomass-derived activated carbons have been developed and assessed recently for their efficacy in removing Cipro from aqueous media. The rice husk-derived activated carbon (RHAC) synthesized in this study demonstrates an excellent balance between surface area (1539.7 m^2^/g) and maximum adsorption capacity (398.4 mg g^−1^), outperforming many previously reported bio-based adsorbents (Table 4). For instance, while bamboo activated with H_3_PO_4_ and K_2_CO_3_ reached the highest values in both metrics (2237 m^2^/g and 613 mg g^−1^, respectively) [45], RHAC offers a more sustainable and environmentally benign synthesis pathway through KOH activation, without compromising adsorption performance.

Beyond its favorable surface characteristics, RHAC also demonstrates remarkably rapid adsorption kinetics. Equilibrium was achieved within just 15 min, significantly faster than many other biomass-based adsorbents. For example, activated carbon derived from *Syagrus romanzoffiana* (Jerivá) achieved equilibrium only after 360 min, despite its high surface area (1435 m^2^/g) and adsorption capacity (335.8 mg g^−1^) [46]. Similarly, activated carbon synthesized from *Albizia lebbeck* seed pods using microwave-assisted KOH activation reached equilibrium in approximately 90 min, with a capacity of 131.14 mg g^−1^ [47]. *Theobroma grandiflorum* (Cupuaçu) bark activated with H_3_PO_4_ exhibited a surface area of 1335.66 m^2^/g and an extremely low capacity of 6.02 mg g^−1^, with an equilibrium time of 266.4 min [48]. Activated carbon derived from kiwi peels, with a surface area of only 0.5 m^2^/g and a maximum capacity of 40.0 mg g^−1^, reached equilibrium in 60 min [49]. Additionally, bamboo activated with Na_2_SiO_3_ indicated low adsorption performance, with a surface area of 312.7 m^2^/g, 17.12 mg g^−1^ capacity, and a 60 min equilibrium time [50]. These comparisons clearly highlight RHAC’s superior kinetics, making it well-suited for time-sensitive water treatment applications.

Regarding adsorption selectivity, RHAC demonstrated a strong affinity for Cipro even in the presence of co-existing organic and inorganic species commonly found in real wastewater. This selectivity likely stems from favorable interactions between RHAC’s surface functional groups and the fluoroquinolone moiety of Cipro, underlining the material’s potential utility in complex environmental matrices.

A notable advantage of RHAC lies in its economic feasibility and environmentally sustainable production pathway. Rice husks are widely available agricultural residues, and using KOH for activation avoids the harsh chemical treatments required by ZnCl_2_- or H_3_PO_4_-based methods, which often necessitate subsequent neutralization and generate environmentally hazardous waste. For example, Jerivá-derived carbon utilized ZnCl_2_ activation [47], while Cupuaçu bark and bamboo-based carbons were activated with H_3_PO_4_ [45,50]. In contrast, KOH activation provides a scalable, low-cost, and less hazardous route for producing high-performance activated carbon.

RHAC’s high surface area and adsorption capacity, rapid kinetic performance, selective binding capabilities, and environmentally responsible production process make it a promising candidate for practical Cipro removal from water. These attributes are particularly valuable for developing sustainable water purification technologies in low-resource settings, where cost, scalability, and environmental impact are critical factors.

In future studies, the adsorption performance of RHAC may be further enhanced by systematically optimizing key synthesis parameters such as the carbonization temperature, KOH-to-biomass ratio, activation duration, and heating rate. Several reports have demonstrated that slight adjustments in these variables can significantly affect surface functionality, pore distribution, and overall adsorption efficiency (Zhang et al., 2017; Ripanda et al., 2024; Yasmin et al., 2024) [14,30,31]. Moreover, post-synthesis surface modifications—such as amination or magnetic functionalization—could be employed to introduce selective binding sites or improve reusability. These strategies offer promising directions for tailoring RHAC properties to meet specific environmental remediation needs.

**Table 4:** Results of some studies in recent years on Cipro adsorption with activated carbon obtained from biomass.

Activated Carbon Source	Activation Process	Surface Area of Adsorbent (m^2^/g)	Maximum Adsorption Capacity (mg·g^−1^)	Equilibrium Time	Reference
Cupuaçu (*Theobroma grandiflorum*) bark	H_3_PO_4_	1335.66	6.02	266.4 min	[48]
Kiwi peels	-	0.5	40.0	60 min	[49]
Jerivá (Syagrus romanzoffiana)	ZnCl_2_	1435	335.8	360 min	[46]
Albizia lebbeck seed pods	Microwave-assisted KOH activation	1824.88	131.14	90 min	[47]
Bamboo	Na_2_SiO_3_	312.7	17.12	60 min	[50]
Bamboo	H_3_PO_4_ and K_2_CO_3_	2237	613	5 day	[45]
Rice husks	KOH	1539.7	398.4	15 min	This work

## 3. Materials and Methods

### 3.1. Materials

Rice husks were supplied by a company located in Samsun province of Turkey. Sigma–Aldrich (Taufkirchen, Germany) provided ciprofloxacin hydrochloride, potassium hydroxide (KOH, ACS reagent, ≥85%), and hydrochloric acid (HCl, ACS reagent, 37%). In the adsorption investigations, laboratory synthesis was employed to create the activated carbon used as an adsorbent. The pure water used in the adsorption tests had a conductivity of 18.2 MΩ·cm, and all other compounds employed were of analytical quality.

### 3.2. Activated Carbon Production from Rice Husks

The process was adapted from the literature [9]. A rice husk sample was ground in a mortar, 150 g of which was taken and placed in a steel tub. The steel tub was placed in a steel reactor with a nitrogen gas flow of 100 mL/min and set at 500 °C for 1 h, increasing by 10 °C per minute, and the carbonization process was carried out. In producing activated carbon from biomass by a chemical activation method, ZnCI_2_, H_3_PO_4_, K_2_CO_3_, and KOH are the most commonly preferred chemical reagents. When KOH is selected from among these reagents, superactive activated carbon with a high surface area can be obtained [51]. Therefore, KOH was preferred as the chemical reagent in this study.

Subsequently, 25 g of the carbonized sample was weighed, and 75 g of KOH was added, dissolved in pure water, and mixed. The steel tub was kept in a 200 °C oven for approximately 30 min to accelerate the dissolution. It was then placed in a steel reactor set at 800 °C for 1 h, increasing by 10 °C per minute, and the activation process was carried out [9].

After activation, the cooled sample was decanted several times with an HCl solution to remove the chlorine. The sample was repeatedly rinsed with deionized water until the filtrate became colorless. After the solution was filtered with filter paper, the rice husk activated carbon (RHAC) collected on the filter was dried in an oven at 105 °C.

### 3.3. Adsorbent Characterization

The surface functional groups of the adsorbent were characterized using Fourier transform infrared spectroscopy (FT-IR). The adsorbent’s surface area measurement and morphological characterization were performed using Brunauer–Emmett–Teller (BET) analysis and scanning electron microscopy (SEM). Furthermore, the adsorbent’s detailed characterization was completed using differential thermal analysis—thermo-gravimetric analysis (DTA/TGA).

### 3.4. Adsorption/Desorption Experiments

The adsorption process was executed in a batch system. Analytical grade Cipro was used directly as the adsorbate in the adsorption investigations without being purified first. The adsorption of Cipro by RHAC in aqueous solutions was investigated. Within the scope of this study, the effects of the contact time, pH, adsorbent dose, and temperature were studied.

After being made and diluted to the necessary starting concentrations, a Cipro stock solution (2000 mg·L^−1^) was used. The contact time study was conducted for ten different durations between 1 and 45 min. Experiments were carried out by taking 50, 75, 100, 150, and 200 mg of RHAC to investigate the effect of the adsorbent amount. For the effect of pH on adsorption, Cipro solutions were prepared with pHs between 2.0 and 8.0 using 0.1 M HCl and 0.1 M NaOH solutions. Cipro solutions were prepared and studied at 298 K, 303 K, and 318 K to determine how temperature affects adsorption. After adsorption, the samples were centrifuged at 4100 rpm for 3 min. Then, 2 mL of the liquid sample remaining in the upper phase due to the sedimentation of the adsorbent was taken, and the absorbance was read at 280 nm wavelength on a UV–VIS spectrophotometer (UV-VIS 754, Shanghai Precision & Scientific Instrument Co., Ltd., Shanghai, China).

(3) $$q_{e}=\frac{\left(C_{o}-C_{e}\;\right)V}{m}$$

In Equation (2), *C_o_* is the starting concentration of Cipro in solution (mg·L^−1^); *C_e_* is the Cipro concentration in solution (mg·L^−1^); V is the volume of the solution (L), and *m* is the weight of RHAC (g).

A series of five consecutive adsorption/desorption cycles was conducted to evaluate the reusability of RHAC for Cipro (50 mg·L^−1^) adsorption. In each cycle, 100 mg of RHAC adsorbed Cipro under optimized conditions. Following the adsorption step, the spent adsorbent was subjected to a desorption treatment using 100 mL of 0.1 M NaOH solution under gentle shaking (150 rpm) for 2 h at room temperature. After desorption, the RHAC samples were thoroughly washed with deionized water until a neutral pH was achieved. The samples were then dried at 60 °C for 12 h before being reused in the next adsorption cycle. The adsorption efficiency in each cycle was calculated by measuring the residual Cipro concentration in the solution using UV–Vis spectrophotometry. This procedure was repeated for five cycles to assess the regeneration potential and stability of RHAC under alkaline desorption conditions.

Additionally, adsorption experiments were performed using hospital wastewater to assess the applicability of RHAC under realistic environmental conditions. The wastewater sample was obtained from a surface water body near a hospital effluent discharge point and then diluted with distilled water at a 1:10 (*v*/*v*) ratio to simulate moderately impacted surface water conditions. Cipro solutions at two different initial concentrations (50 mg·L^−1^ and 250 mg·L^−1^) were prepared using this diluted matrix. All adsorption experiments were conducted under this study’s previously optimized experimental conditions, including contact time, adsorbent dosage, pH, and temperature. These experiments were designed to compare adsorption behavior in a realistic wastewater background with that observed in distilled water systems, without altering the operational parameters.

## 4. Conclusions

This study highlights the successful synthesis of high-performance activated carbon (RHAC) from rice husks using potassium hydroxide activation, achieving a substantial surface area of 1539.7 m^2^/g and a maximum Cipro adsorption capacity of 398.4 mg·g^−1^. The adsorption process exhibited strong pH dependence, with optimum removal efficiency at pH 3.0 due to favorable electrostatic interactions. Kinetic studies showed that the adsorption followed a pseudo second-order model, indicating that chemisorption was the dominant mechanism, characterized by electron sharing or exchange between adsorbent and adsorbate. This finding was further supported by the close match between experimental and modeled equilibrium capacities and a high correlation coefficient (R^2^ = 0.9981). Thermodynamic analysis confirmed the endothermic and spontaneous nature of the adsorption. Among the tested isotherm models, the Freundlich model provided the best fit, implying a multilayer adsorption process on a heterogeneous surface. Overall, RHAC emerges as a cost-effective, sustainable, and high-capacity adsorbent for antibiotic removal, promoting the valorization of agricultural waste and supporting environmentally friendly water treatment strategies. Although the RHAC produced in this study presented an excellent adsorption capacity and rapid kinetics, further studies are recommended to systematically investigate the influence of carbonization temperature, KOH dosage, activation duration, and other synthesis parameters. Future studies focusing on synthesis parameter optimization could further enhance the adsorptive performance and selectivity of the RHAC materials.

## Figures and Tables

**Table 1:** Adsorption parameters.

Isotherm	Parameters	Temperature (K)		
		**298**	**303**	**318**
Langmuir	q_m_ (mg g^−1^)	2272.28	2329.55	2345.43
	K_L_ (L mg^−1^)	0.1060	0.1195	0.1371
	R^2^	0.9631	0.9737	0.9833
Freundlich	K_F_ [(mg g^−1^)(L mg^−1^)]^1/n^	436.43	459.84	474.33
	1/n	0.3856	0.3909	0.3856
	n	2.59	2.55	2.50
	R^2^	0.9933	0.9970	0.9966
Temkin	B (J mol^−1^)	6.18	6.19	6.24
	K_T_ (L mg^−1^)	1.9996	2.1779	2.2162
	R^2^	0.9339	0.9463	0.9663
Dubinin–Radushkevich	q_m_ (mg g^−1^)	243.57	234.17	219.39
	E (kJ mol^−1^)	1.02	1.14	1.22
	R^2^	0.8248	0.8295	0.8395

**Table 2:** Parameters of kinetic models.

**Model**	***q_exp_*** **(mg·g^−1^)**	***q_e_*** **(mg·g^−1^)**	***k***	**R^2^**
Pseudo first order	95.1	45.56	0.1174	0.8862
Pseudo second order		100.15	0.0051	0.9981

**Table 3:** Calculated thermodynamic parameters for the adsorption of Cipro on RHAC.

T (K)	ΔG° (kJ·mol^−1^)	ΔH° (kJ·mol^−1^)	ΔS° (J·mol^−1^·K^−1^)
298	−9.1295	6.6114	52.9223
303	−9.7529		
318	−10.1838		

## Data Availability

The original contributions presented in this study are included in the article/Supplementary Material. Further inquiries can be directed to the corresponding authors.

## Supplementary Materials

The following supporting information can be downloaded at: https://www.mdpi.com/article/10.3390/molecules30122501/s1, Figure S1: Thermodynamic graphs at 298 K (a), 303 K (b), 318 K (c). Figure S2: Freundlich graphs at 298 K (a), 303 K (b), 318 K (c). Figure S3: Temkin graphs at 298 K (a), 303 K (b), 318 K (c). Figure S4: Dubinin-Radushkevich (D-R) graphs at 298 K (a), 303 K (b), 318 K (c). Figure S5: Thermodynamic graphs at 298 K (a), 303 K (b), 318 K (c). Figure S6: Van’t Hoff graph. References [2,5,6,9,52,53,54,55,56] are cited in the Supplementary Materials.
