# Supplementary material for: Efficient Removal of Ciprofloxacin from Water Using High-Surface-Area Activated Carbon Derived from Rice Husks: Adsorption Isotherms, Kinetics, and Thermodynamic Evaluation
Source: Molecules. 2025 Jun 7;30(12):2501. doi: 10.3390/molecules30122501 (PMC12196439; doi:10.3390/molecules30122501)
Supplement: Supplementary file 1 [file molecules-30-02501-s001.zip › molecules-3600861-supplementary.pdf]

Supplemental Material

Langmuir isotherm

Equation S1, the linear equation of the Langmuir isotherm (Figure S1), was used to compute it.

$$\frac{C_e}{q_e} = \frac{1}{q_m K_L} + \frac{C_e}{q_m} \quad \text{(S1)}$$

The Langmuir adsorption constant is K_L (L·mg^{-1}), the maximum adsorption capacity is q_m (mg·g^{-1}), the equilibrium concentration is C_e (mg·L^{-1}), and the adsorption quantity at equilibrium is q_e (mg·g^{-1}). According to the Langmuir isotherm hypothesis, the adsorbate is deposited as a monolayer on a homogeneous adsorbent surface [1].

$$R_L = \frac{1}{1 + K_L C_O} \quad \text{(S2)}$$

R_L is a unitless constant, K_L is the Langmuir constant (L·mg^{-1}), and C_0 is the initial concentration of Cipro (mg·L^{-1}). The type of isotherm, whether negative ($R_L > 1$), linear ($R_L = 1$), positive ($0 < R_L < 1$), or irreversible ($R_L = 0$), is indicated by the R_L values [1–3].

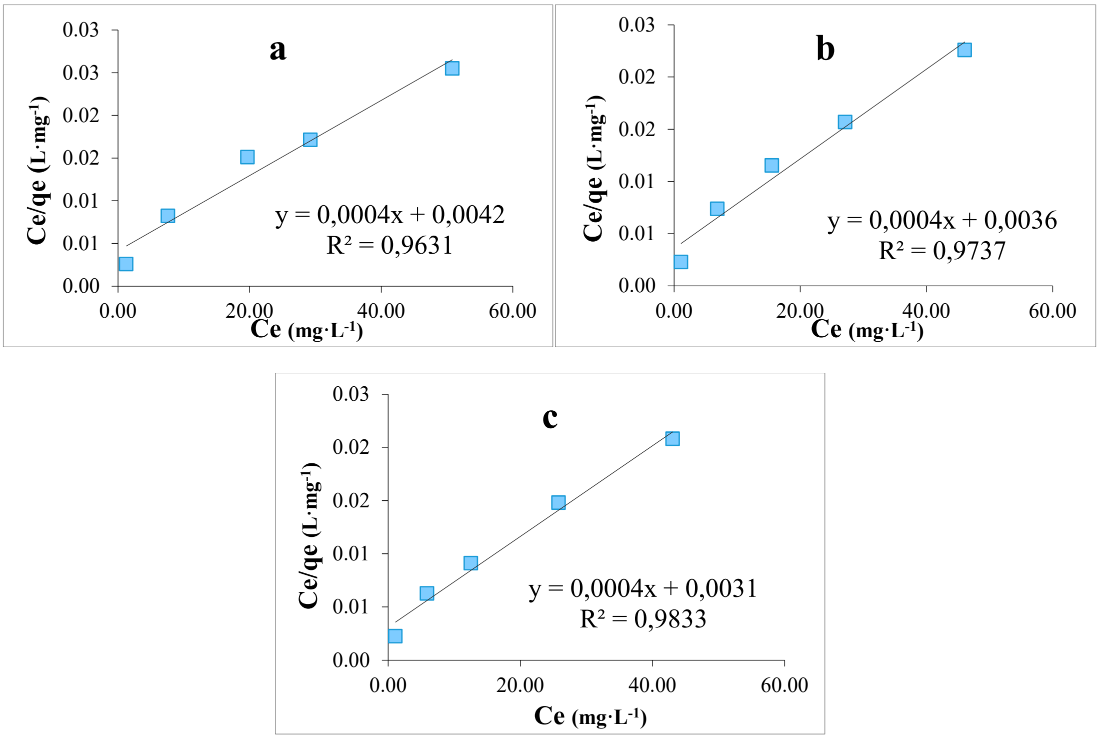

Figure S1. Langmuir graphs at 298K (a), 303K (b), 318K (c).

Freundlich Isotherm

Equation S3 provides the linear equation for the Freundlich isotherm (Figure S2). The terms "adsorption intensity" (n) and "adsorption capacity" (K_F) are used. Freundlich states that the adsorption sites on an adsorbent's surface are heterogeneous, meaning they have several different types [4].

$$lnq_e = lnK_F + \frac{1}{n} lnC_e \qquad (S3)$$

The Freundlich constant (K_F) is related to adsorption capacity within the temperature range under investigation. The adsorption process is endothermic, as seen by the increase in K_F value with temperature [5].

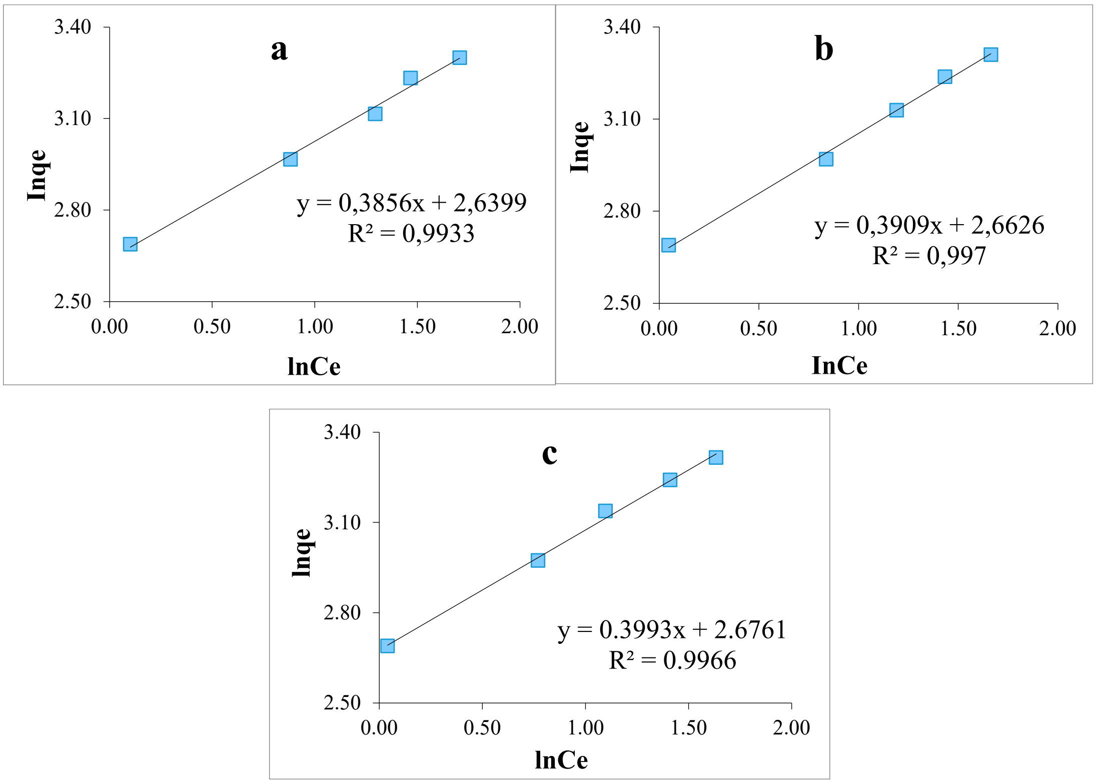

Figure S2. Freundlich graphs at 298K (a), 303K (b), 318K (c).

Temkin Isotherm

The Temkin isotherm (Figure S3) was calculated using equations S4 and S5.

$$q_e = B_T lnK_T + B_T lnC_e \quad (S4)$$

$$B_T = \frac{RT}{b} \quad (S5)$$

B_T ($j \cdot mol^{-1}$): Adsorption heat, K_T ($L \cdot mg^{-1}$): Temporal coefficient and adsorption capacity, T (K): Absolute temperature, R: Universal gas constant ($8.314\ j \cdot mol^{-1} \cdot K^{-1}$).

At 298, 303, and 318 K, respectively, the equilibrium binding constant (K_T) corresponds to the maximal binding energy in the Temkin isotherm. As the temperature rose during the adsorption process, K_T's value increased, indicating a physical bonding capacity between activated carbon and Cipro. B_T is a constant whose value depends on the heat of adsorption. The results are less than 40 j·mol^{-1}, indicating a physical adsorption process [2,6,7].

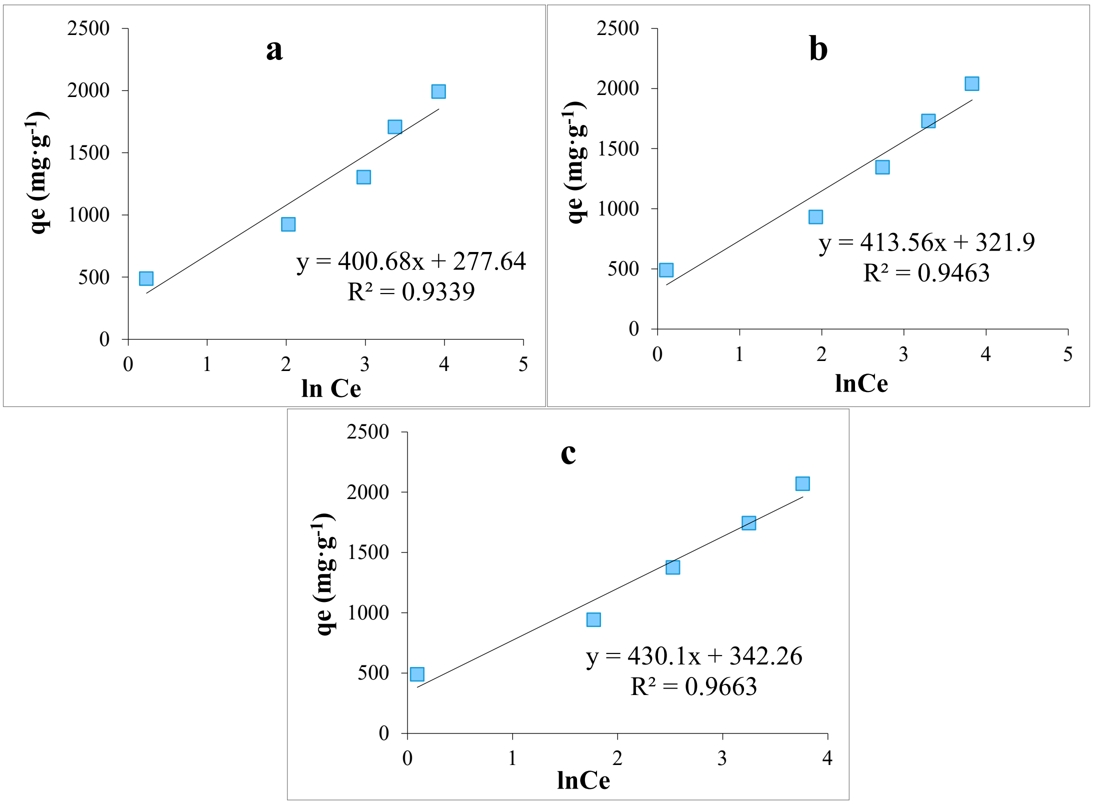

Figure S3. Temkin graphs at 298K (a), 303K (b), 318K (c).

Dubinin-Radushkevich (D-R) Isotherm

Using equations S6-S8, the Dubinin-Radushkevich (D-R) isotherm (Figure S4) was computed.

$$lnq_e = lnq_m - k\varepsilon^2 \quad \text{(S6)}$$

q_e is the amount of dye removed per unit adsorbent (mg·g^{-1}), k is the constant related to the adsorption energy (mol^2·kj^{-2}), q_m is the D-R adsorption capacity (mg·g^{-1}), ε is the polanyi potential (kj·mol^{-1}), T is the temperature (K), and R is the gas constant (kj·K^{-2}·mol^{-2}).

$$\varepsilon = RT \ln\left(1 + \frac{1}{C_e}\right) \quad \text{(S7)}$$

$$E = (2k)^{-1/2} \quad \text{(S8)}$$

Equation S8's adsorption energy E (kj·mol^{-1}) details the adsorption mechanism. The determined E value indicates that one of the adsorption processes is physical, ion exchange, or chemical. In cases where E is less than 8 kj·mol^{-1}, the adsorption process takes place via physical interactions; in the range of 8 < E

< 16 kj·mol^{-1}, ion exchange takes place; and in the case of E values greater than 16 kj·mol^{-1}, chemical interaction takes place [6,8].

Since the adsorption energies (E) are less than 8 kj·mol^{-1}, this result further supports the notion that a physical adsorption process is occurring [2,7,9].

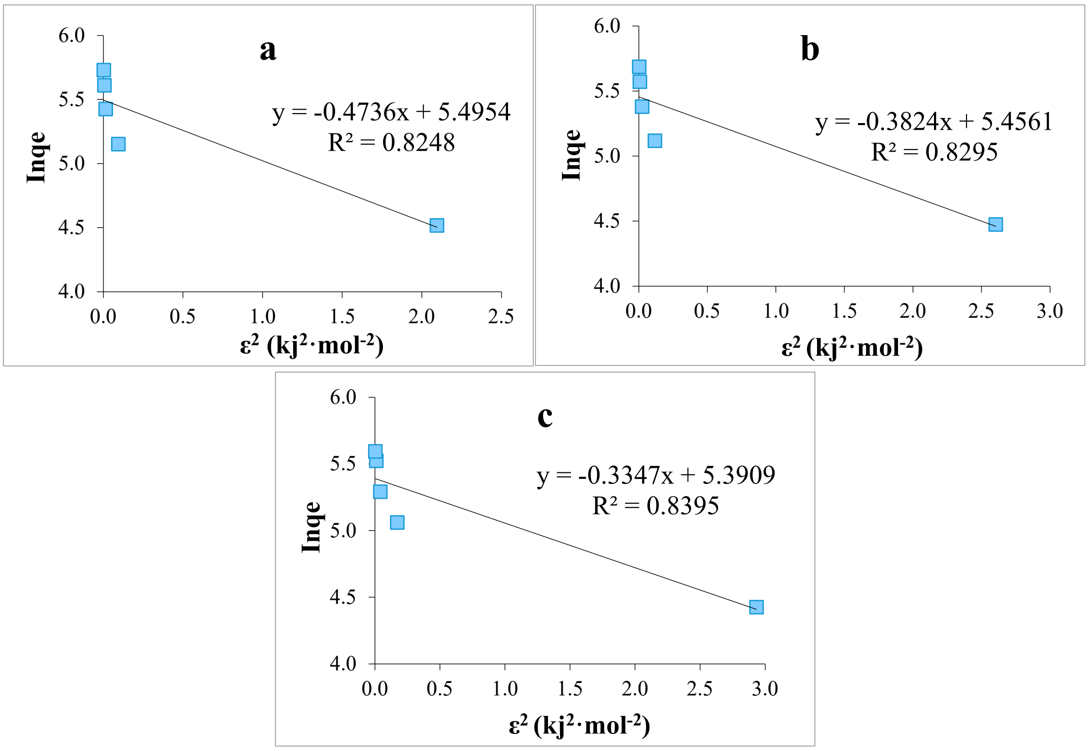

Figure S4. Dubinin-Radushkevich (D-R) graphs at 298K (a), 303K (b), 318K (c).

Thermodynamic

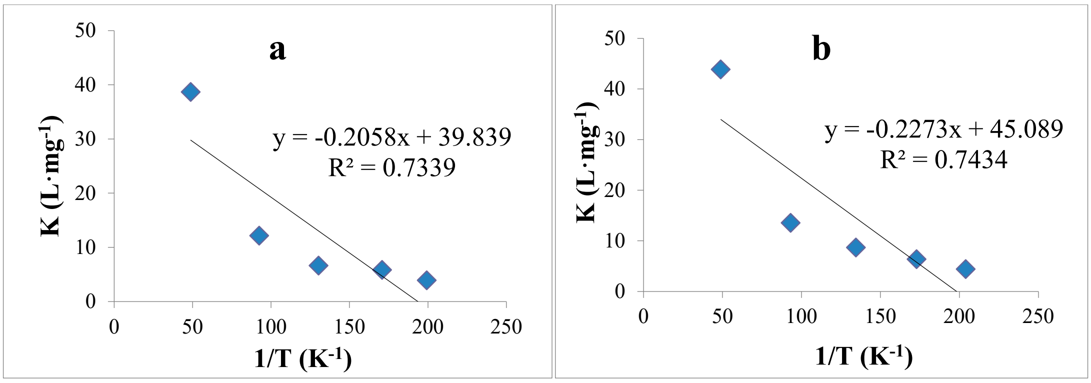

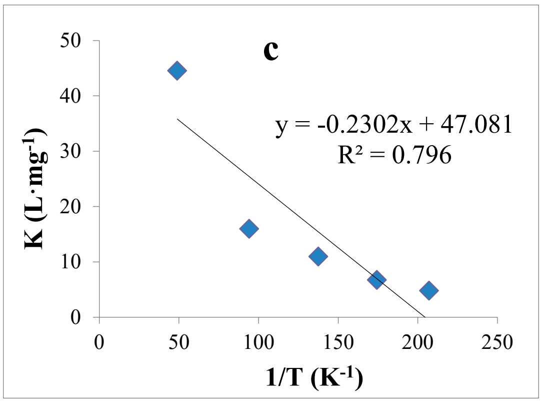

Figure S5. Thermodynamic graphs at 298K (a), 303K (b), 318K (c).

The adsorption process of Cipro on RHAC was investigated at three different temperature values (298K, 303K, 318K) (Figure S5). *K* values were calculated with the help of C_e and q_e values at the equilibrium moment found from the experimental results. (The equilibrium constant *K* (initially calculated in $L{\cdot}mg^{-1}$ from adsorption data) was converted to a dimensionless form by normalizing with a standard reference concentration, ensuring compatibility with the Van't Hoff logarithmic transformation.) *K* values;

$$K = \frac{q_e}{C_e} \quad (S9)$$

It is calculated using equation S9. The *K* values found are used in the Van't Hoff equation.

$$\ln K = -\frac{\Delta H}{R}\frac{1}{T} + \frac{\Delta S}{R} \quad (S10)$$

Equation S10 is linear, and if the $1/T$ values are plotted against the ln*K* values, the adsorption enthalpy $\Delta H°$ is found from the slope of the line, and the entropy value $\Delta S°$ is found from where it intersects the axis [2]. Van't Hoff graph drawn using equation S10 is shown in Figure S6.

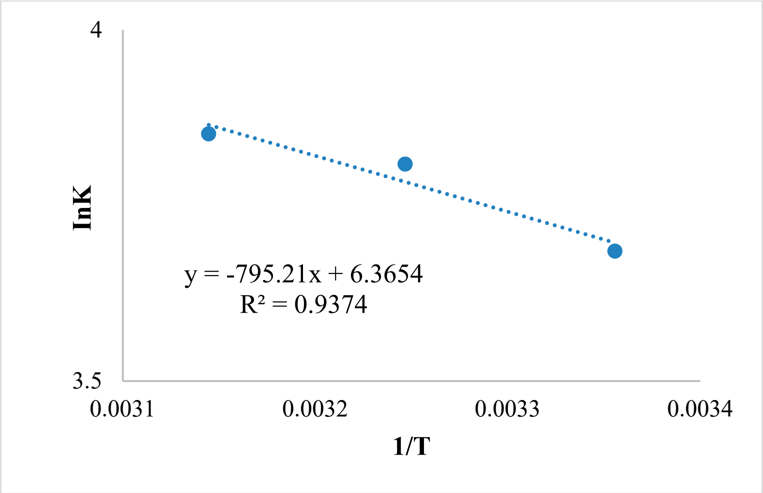

Figure S6. Van't Hoff graph.

The free energy value $\Delta G°$ of the adsorption process is calculated using the K value calculated using the C_e and q_e values at the equilibrium moment.

$$\Delta G° = -RTlnK \quad (S11)$$

The $\Delta H°$ value for Cipro adsorption on RHAC was calculated as 6.6114 kj·mol^{-1}. The adsorption enthalpy is lower than 40 kj·mol^{-1}, indicating that the physical mechanism controls the adsorption processes [9].

The $\Delta S°$ value for Cipro adsorption on RHAC was calculated as 52.9223 kj·mol^{-1}. The positive values of entropy may be due to some structural changes in the adsorbate and adsorbents during the adsorption process from aqueous solution to the adsorbents. Also, the positive value of entropy indicates the increased randomness at the solid-liquid interface during the adsorption of Cipro substance on adsorbents.

The negative $\Delta G°$ values calculated for Cipro adsorption on RHAC at temperatures of 298K, 303K, and 318K indicate that Cipro adsorption on RHAC is feasible and occurs spontaneously without an external energy requirement. In general, a $\Delta G°$ value between "0 and -20 kj·mol^{-1}" indicates that the event is physical adsorption, while a value between "-80 and -400 kj·mol^{-1}" indicates that the event is chemical adsorption. Accordingly, the negative $\Delta G°$ values in Table 2, in the range of -9.1295 and -10.1838 kj·mol^{-1}, show us that physical adsorption is effective [6,8].

The negative $\Delta G°$ values also indicate that the adsorption efficiency increased with increasing temperature; the more ciprofloxacin in the equilibrium solution, the lower the temperature. The results obtained show that the adsorption is spontaneous under the operating conditions studied due to the negative sign of the $\Delta G°$ values increasing as the temperature of the adsorption system increases. Although $\Delta H°$ and $\Delta S°$ were assumed to be constant over the investigated temperature range, the increasingly negative $\Delta G°$ values with rising temperature are in complete agreement with the Gibbs free energy relationship. This confirms that the adsorption process is endothermic and entropy-driven, and aligns well with the Van't Hoff model expectations for physical adsorption. As a result, it can be said that the adsorption process is spontaneous and requires energy to complete, and a higher temperature is better for reaching maximum efficiency.

References

1. Kirec, O.; Alacabey, I.; Erol, K.; Alkan, H. Removal of 17β-Estradiol from Aqueous Systems with Hydrophobic Microspheres. *J. Polym. Eng.* **2021**, *41*, 226–234.
2. Shin, H.S.; Kim, J.H. Isotherm, Kinetic and Thermodynamic Characteristics of Adsorption of Paclitaxel onto Diaion HP-20. *Process Biochem.* **2016**, *51*, 917–924.
3. Caliskan, N.; Kul, A.R.; Alkan, S.; Gokirmak Sogut, E.; Alacabey, I. Adsorption of Zinc(II) on Diatomite and Manganese-Oxide-Modified Diatomite: A Kinetic and Equilibrium Study. *J. Hazard. Mater.* **2011**, *193*, 27–36.
4. Alacabey, I.; Kul, A.; Ece, M.; Alkan, H. Chrome(III) Adsorption on Van Lake Natural Sediment and Modified Sediment (Isotherm and Thermodynamic Analysis Study). *DÜMF Eng. J.* **2020**, *11*, 1225–1232.
5. Tegin, I.; Demirel, M.F.; Alacabey, I.; Yabalak, E. Investigation of the Effectiveness of Waste Nut Shell–Based Hydrochars in Water Treatment: A Model Study for the Adsorption of Methylene Blue. *Biomass Convers. Biorefin.* **2022**, 1–14.

6. Alacabey, I. Antibiotic Removal from the Aquatic Environment with Activated Carbon Produced from Pumpkin Seeds. *Molecules* **2022**, *27*, 1380.
7. Lee, J.J. Isotherm, Kinetic and Thermodynamic Characteristics for Adsorption of Congo Red by Activated Carbon. *Korean Chem. Eng. Res.* **2015**, *53*, 64–70.
8. Alacabey, I. Adsorptive Removal of Cationic Dye from Aqueous Solutions Using Bardakçı Clay. *Int. J. Agric. Environ. Food Sci.* **2022**, *6*, 80–90.
9. Alacabey, I. Endosulfan Elimination Using Amine-Modified Magnetic Diatomite as an Adsorbent. *Front. Chem.* **2022**, *10*, 907302.
